# Activators of Nrf2 to Counteract Neurodegenerative Diseases

**DOI:** 10.3390/antiox12030778

**Published:** 2023-03-22

**Authors:** Rosa Amoroso, Cristina Maccallini, Ilaria Bellezza

**Affiliations:** 1 Department of Pharmacy, University “G.d’Annunzio” of Chieti-Pescara, Via dei Vestini, 31, 66100 Chieti, Italy; rosa.amoroso@unich.it; 2 Department of Medicine and Surgery, University of Perugia, Polo Unico Sant’Andrea delle Fratte, P.e Lucio Severi 1, 06132 Perugia, Italy; ilaria.bellezza@unipg.it

**Keywords:** Alzheimer’s disease, antioxidant response, electrophilic activators, multitargeting compounds, Nrf2 inducers, protein–protein interaction inhibitors, oxidative stress, Parkinson’s disease

## Abstract

Neurodegenerative diseases are incurable and debilitating conditions that result in progressive degeneration and loss of nerve cells. Oxidative stress has been proposed as one factor that plays a potential role in the pathogenesis of neurodegenerative disorders since neuron cells are particularly vulnerable to oxidative damage. Nuclear factor (erythroid-derived 2)-like 2 (Nrf2) is strictly related to anti-inflammatory and antioxidative cell response; therefore, its activation and the consequent enhancement of the related cellular pathways have been proposed as a potential therapeutic approach. Several Nrf2 activators with different mechanisms and diverse structures have been reported, but those applied for neurodisorders are still limited. However, in the very last few years, interesting progress has been made, particularly in enhancing the blood–brain barrier penetration, to make Nrf2 activators effective drugs, and in designing Nrf2-based multitarget-directed ligands to affect multiple pathways involved in the pathology of neurodegenerative diseases. The present review gives an overview of the most representative findings in this research area.

## 1. Introduction

Neurodegenerative diseases affect millions of people worldwide and are a leading cause of disability and a major cause of mortality [1]. The treatment of these pathological conditions is only palliative; therefore, there is an urgent need for effective therapeutic agents, as well as a deeper understanding of the molecular changes affecting neuronal cells during the disease progression. Neuroinflammation is a hallmark in the development of neurodegenerative diseases, as well as nitroxidative stress, which is due to the unbalanced production of both reactive oxygen species (ROS) and reactive nitrogen species (RNS) [2,3]. The key role played by ROS in the onset of age-related neurodegenerative diseases indicates that erythroid-derived 2-like 2 (Nrf2), the master regulator of redox homeostasis [4], may be a promising target for therapeutic interventions. Several Nrf2 activators with different mechanisms and diverse structures have been reported in the literature, but those applied to neurodegenerative diseases are still limited [5]. However, in the very last few years, interesting progress has been made in this field, particularly in enhancing the blood–brain barrier (BBB) permeability of Nrf2 activators, to make them effective drugs, and in designing Nrf2-based multitarget-directed ligands to affect multiple pathways involved in the pathology of neurodegenerative diseases. In the present review, the implications of oxidative stress and Nrf2 activation in the therapy of neurodegeneration are revised, with a particular focus on Alzheimer’s disease (AD) and Parkinson’s disease (PD), which are the two most prevalent age-related neurodegenerative diseases [1]. Moreover, progress in the development of new Nrf2 activators able to counteract neuroinflammation is discussed.

## 2. Implication of Oxidative Stress and Nrf2 Activation in Neurodegenerative Diseases

**Figure 1:**
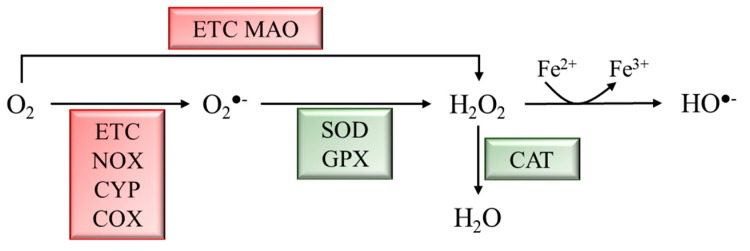
Schematic representation of ROS generation and scavenging. Molecular oxygen (O_2_) can be partially reduced to superoxide anion (O_2_^●−^) by the ETC, NADPH oxidases (NOX), cytochrome P450 (CYP) oxidases, and cyclooxygenases (COX), or to hydrogen peroxide (H_2_O_2_) by the ETC and monoamine oxidases (MAO). H_2_O_2_ can be converted into hydroxyl radical (HO^●−^) through the Fenton rection. Enzymatic antioxidants scavenge ROS to produce less reactive molecules. Superoxide dismutase (SOD) and glutathione peroxidase (GPX) convert O_2_^●−^ to H_2_O_2_, which is converted into H_2_O by catalase (CAT).

ROS are a byproduct of several cellular metabolic pathways and enzymatic reactions, and they are classified as radicals, including superoxide anion (O_2_^●−^) and hydroxyl (HO^●−^), as well as nonradical species such as hydrogen peroxide (H_2_O_2_) [6]. ROS are reactive molecules with a very short half-life. H_2_O_2_, the most stable ROS, has a cellular half-life of 10^−3^ s, 1000 times higher than other ROS [7,8]. Although ROS production might be due to environmental factors, it mainly derives from metabolic activities. During mitochondrial respiration, in the electron transport chain (ETC), approximately 1–2% of O_2_ is not reduced to water, leading to the generation of O_2_^●−^ and H_2_O_2_. Moreover, cytosolic oxidoreductases such as NADPH oxidases (NOX), cytochrome P450 (CYP) oxidases, cyclooxygenases (COX), and monoamine oxidases (MAO) may contribute to ROS production [8]. Physiological ROS levels are maintained by a plethora of exogenous and endogenous antioxidant defenses. Among the exogenous antioxidants, ascorbic acid (vitamin C), α-tocopherol (vitamin E), and carotenoids play a pivotal role. Endogenous antioxidants include enzymatic antioxidants, e.g., superoxide dismutase (SOD), glutathione peroxidase (GPX), and catalase (CAT), as well as nonenzymatic scavengers, e.g., glutathione (GSH) (Figure 1) [9].

Redox homeostasis should be strictly controlled since ROS play fundamental biological roles in guaranteeing redox signaling, a transduction system in which reversible electron transfer reactions involving ROS to effector target proteins culminate in the regulation of numerous physiological functions, including neuronal development and function, cellular proliferation and differentiation, and aging prevention [10]. For example, due to its ability to cross the phospholipidic bilayer of the cellular membrane, H_2_O_2_ can act in both an autocrine and a paracrine manner [7]. When the production of ROS exceeds cellular detoxification capacity, redox balance gets compromised, and oxidative stress insurges. In these conditions, high ROS levels can oxidize nucleic acids, proteins, and lipids, thus leading to cell dysfunction and eventually cell death [9,11].

**Figure 2:**
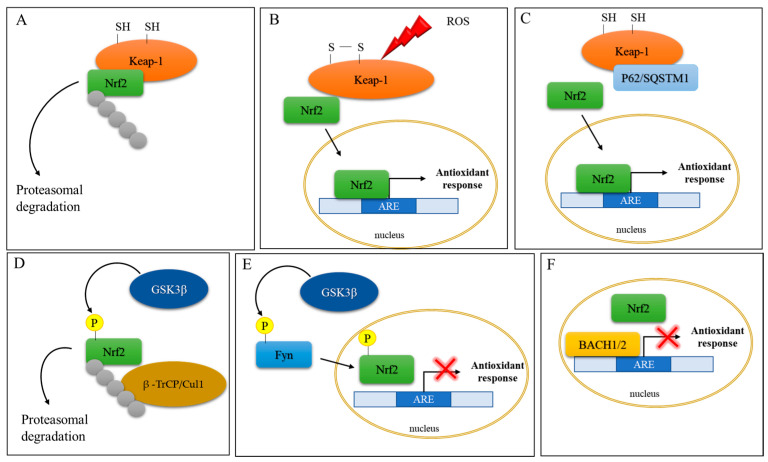
Schematic representation of Nrf2 activation. (**A**) Under basal conditions, Nrf2 activity is controlled by the cytoplasmic repressor protein Keap1, which sequesters Nrf2 in the cytosol, leading to its ubiquitination and proteasomal degradation. (**B**) Under oxidative stress conditions, two of the Cys residues in Keap1 become oxidized, impeding the correct orientation of Nrf2 and inhibiting its ubiquitination and degradation. The newly synthesized Nrf2 translocates to the nucleus to induce the expression of antioxidant response genes. (**C**) P62/sequestosome 1 (p62/SQSTM1) competes with Nrf2 for Keap-1 binding and leads to Nrf2 activation and nuclear translocation. (**D**) Glycogen synthase kinase-3β (GSK-3β) phosphorylates Nrf2, which is in turn ubiquitinated by β-transducin repeat-containing protein/Cullin-1 E3 ubiquitin ligase (β–TrCP/Cul1). (**E**) GSK-3β phosphorylates the protein kinase Fyn, which phosphorylates Nrf2, leading to its nuclear export and culminating in inhibition of gene expression (red cross). (**F**) The BTB and CNC homology transcription factors (BACH1 and BACH2) repress Nrf2 activity (red cross) by competing for ARE binding.

The master regulator of redox homeostasis is erythroid-derived 2-like 2 (Nrf2) [4]. Nrf2 is a cap ‘n’ collar (CNC) basic leucine zipper (bZIP) transcription factor responsible for the expression of genes containing the antioxidant responsive element (ARE) sequence in their promoter region, including genes linked to the synthesis or use of GSH [12]. Under basal conditions, Nrf2 has a rapid turnover, with a half-life of approximately 20 min. In fact, Nrf2 activity is controlled by a cytoplasmic repressor protein Keap1, which sequesters Nrf2 in the cytosol and, by recruiting CUL3-dependent E3-ubiquitin ligase, leads to its ubiquitination and proteasomal degradation [13,14]. Under oxidative stress conditions, two of the 27 Cys residues in Keap1 become oxidized, causing a conformational change that, according to the “hinge and latch” model [15,16], impedes the correct orientation of Nrf2 and inhibits its ubiquitination and degradation [13]. The newly synthesized Nrf2 can in turn translocate to the nucleus to exert its functions [12]. Outside of Keap-1, Nrf2 activity is also controlled by other regulators. For example, glycogen synthase kinase-3β (GSK-3β) phosphorylates Nrf2, aiding its ubiquitination by β-transducin repeat-containing protein (β -TrCP)/Cullin-1 E3 ubiquitin ligase in a Keap-1-independent manner [17]. GSK-3β also phosphorylates the protein kinase Fyn, which translocates into the nucleus where it phosphorylates Nrf2, leading to its nuclear export and ubiquitin-dependent proteasomal degradation [18]. To add a further layer of complexity, the BTB and CNC homology transcription factors (BACH1 and BACH2) repress Nrf2 activity by competing for ARE binding [19]. On the other hand, p62/sequestosome 1 (p62/SQSTM1), a ubiquitin-binding protein, competing with Nrf2 for Keap-1 binding, leads to Nrf2 stabilization and, hence, activation (Figure 2) [20].

Due to the high oxygen consumption, the presence of polyunsaturated fatty acids, and auto-oxidation of neurotransmitters (e.g., dopamine), the brain is highly vulnerable to ROS-mediated damage [21]. Thus, any imbalance of redox homeostasis could affect brain cells. For example, aging and age-related diseases such as neurodegenerative disorders have been linked to the progressive dysfunction of redox control mechanisms [22]. The disruption of redox balance during healthy aging correlates with the finding that Nrf2 activity in brain decreases with age [23]. Brain expression of Nrf2 is higher in glial cells (astrocytes and microglia) than in neurons, suggesting that glial cells can be causally involved in neurodegenerative diseases despite not being the cells primarily affected by the disease. This concept, known as a non-cell-autonomous mechanism, is recognized to essentially contribute to neurodegeneration. It has been proposed that microglia and astrocytes causally participate in the pathogenesis and progression of several neurodegenerative diseases [24,25].

Microglial cells behave as the innate immune cells of the CNS [26]. Resting microglial cells have a ramified morphology; however, when activated by a danger signal, they undergo a morphological change, becoming amoeboid. While resting microglial cells continuously monitor brain activities to maintain homeostasis, activated microglial cells release cytokines and proinflammatory mediators to eliminate the threat. This acute response of microglial cells is protective, but overactivation of microglia, by inducing severe oxidative stress and neuroinflammation, leads to many neurodegenerative diseases [26]. In aged microglial cells, migration ability and phagocytosis are compromised, and the release of proinflammatory cytokines lasts for prolonged periods, strongly contributing to neurodegeneration [27]. Disturbance of Nrf2 activity and, hence, increased oxidative stress have been linked to several neurodegenerative diseases such as Parkinson’s disease and Alzheimer’s disease.

### 2.1. Nrf2 and Parkinson’s Disease

PD is a progressive neurological movement disorder characterized by tremor, bradykinesia, rigid muscles, and loss of postural balance [28]. The clinical feature of PD is the depletion of dopaminergic (DArgic) neurons in the substantia nigra pars compacta (SNpc), which results in dopamine (DA) deficiency [29]. PD is prevalently an idiopathic disease, with only 10–15% of cases having a family history. However, Lewy bodies found in both sporadic and familial cases of PD contain aggregated forms of α-synuclein (α-syn). The α-syn oligomers are cytotoxic to DArgic neurons [30]. The toxic effects of α-syn oligomers can be ascribed to α-syn enzymatic ferrireductase activity. Aggregation indeed inhibits α-syn enzymatic activity, leading to an accumulation of oxidized Fe, which can participate in the Fenton reaction, culminating in oxidative stress induction [31]. Moreover, mitochondrial α-syn accumulation can suppress mitochondrial respiratory complex I, leading to ROS production and, thus, to oxidative stress [32]. The crucial role of mitochondria-generated ROS in PD pathogenesis was determined from the finding that the 1-methyl-4-phenyl 1,2,3,6-tetrahydropyridine (MPTP) metabolite 1-methyl-4-phenylpyridinium (MPP^+^) hampers mitochondrial complex I, causing electron leakage and ROS production [33]. PD-like symptoms indeed arose in individuals using illegal drugs contaminated by MPTP, and brain postmortem examinations revealed damage to the DArgic neurons in the SNpc.

Other PD genetic risk factors comprise genes involved in the control of mitochondrial redox balance including Parkin, PTEN-induced kinase 1 (PINK1), parkinsonism-associated deglycase (PARK7), and leucine-rich repeat serine/threonine protein kinase 2 (LRRK2) [34]. Parkin, an E3 ubiquitin ligase, and PINK1, a serine/threonine kinase, form a signal transduction pathway exerting a pivotal role in the removal of damaged mitochondria by the mitophagic process [35]; PARK7 encodes for DJ-1, a protein that, by binding the mitochondrial complex I, improves its activity [36]. Mutations in PINK1, DJ-1, and LRRK2 result in mitochondrial dysfunction with impaired bioenergetics and lead to uncontrolled ROS generation [36,37]. It should be emphasized that DA metabolism itself is a source of oxidative stress. In fact, excess DA can be catabolized by monoamine oxidase (MAO), producing H_2_O_2_ or undergoing auto-oxidation to produce highly reactive dopamine quinones (DAQs) and semiquinones [38].

**Figure 3:**
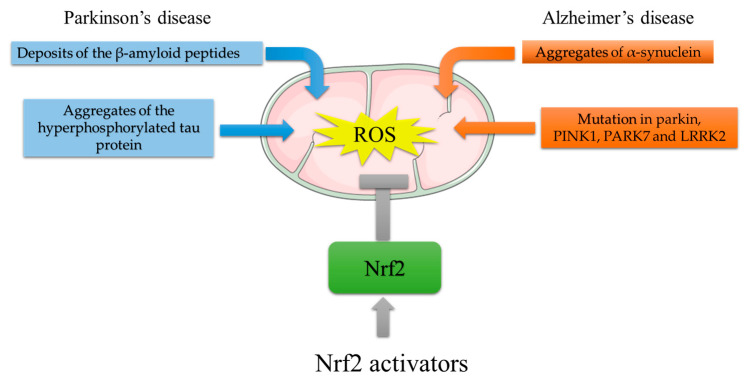
Mitochondrial ROS generation by PD and AD risk factors, and protective effects of Nrf2 activating compounds. ROS generation in AD: deposition of Aβ plaques induces mitochondrial stress; aggregates of the hyperphosphorylated tau protein impair mitochondria distribution and function. ROS generation in PD: aggregated α-syn accumulation suppresses mitochondrial respiratory complex I; mutations in Parkin, PTEN-induced kinase 1 (PINK1), parkinsonism-associated deglycase (PARK7), and leucine-rich repeat serine/threonine protein kinase 2 (LRRK2) result in mitochondrial dysfunction with impaired bioenergetics. The Nrf2-mediated increase in antioxidant response genes can reduce ROS burst. In this light, Nrf2 activators might counteract the ROS-induced damage that leads to neurodegeneration.

The crucial role played by ROS in PD indicates Nrf2 implication in this disease. In vivo studies showed that both treatment with MPTP and overexpression of α-syn increased DArgic cell death in Nrf2 KO mice [39,40]. Moreover, the Nrf2-induced cytoprotective gene NQO1 (NADPH-quinone oxidoreductase) is partially sequestered in Lewy bodies, together with p62/SQSTM1, a known activator of Nrf2 [41]. Moreover, Bach1 knockout protects mice against MPTP-induced dopaminergic cell death [42]. Moreover, the brain of LRRK2-transgenic mice and the LRRK2-overexpressing neuronal cell line show reduced expression of Nrf2 and its target genes via GSK3β activation [43]. Studies in Drosophila showed that Nrf2 activation induces mitophagy and counteracts neuronal degeneration in Parkin/Pink1 knockdown flies [44]. In addition to direct neuronal toxicity, α-syn oligomers can activate microglial cells promoting neuroinflammation, which, by further increasing ROS production via NADPH oxydase 2 (NOX2) activation, augments oxidative stress [34,41]. In this context, DJ-1, by interacting with the p47phox subunit of NOX2, inhibits its action, reducing ROS production [45]. From all the above, PD genetic risk factors are connected to aberrant Nrf2 signaling which might contribute to oxidative stress (Figure 3).

### 2.2. Nrf2 and Alzheimer’s Disease

Alzheimer’s disease (AD) is the prevalent type of dementia in the elderly population. AD is characterized by memory loss and deterioration of other cognitive functions including comprehension, judgment, and orientation due to synaptic loss and selective neuronal death [46]. AD brains are characterized by the accumulation of extracellular senile plaques composed of deposits of β-amyloid peptides Aβ (1–40) and Aβ (1–42), as well as intracellular neurofibrillary tangles (NFTs) composed of aggregates of the hyperphosphorylated tau protein [40]. Most AD cases are classified as sporadic late-onset AD (onset after 65 years of age), whereas only 5% are autosomal-dominant early-onset AD [47,48]. Early-onset AD is caused by a mutation in three proteins involved in the amyloidogenic pathway: APP (amyloid precursor protein), γ-secretase presenilin 1 (PSEN1), and presenilin 2 (PSEN2). The key role played by ROS in aging and the fact that idiopathic AD is an age-related disease strongly suggest that ROS are involved in AD pathogenesis. This hypothesis is corroborated by the finding that Aβ binds to mitochondrial membranes, thus affecting mitochondrial function and accelerating ROS production [49]. The pivotal role of ROS in AD is based on the findings that Aβ (1–42) induces mitochondrial stress accompanied by ROS generation in AD patients [50,51], and that Aβ plaque deposition leads to oxidative stress, which, propagating spatially over time, leads to neuronal cell death [52,53]. Not only Aβ, but also tau protein is connected to mitochondrial ROS generation in AD. For example, compared to wildtype mice, tau knockout presented reduced oxidative damage, improved memory skills, and improved mitochondrial bioenergetics [54]. Furthermore, ROS induce tau polymerization [55], and tau overexpression induces oxidative stress in vitro and in mice [56,57]. Moreover, Nrf2 activation prevents tau protein aggregation in transgenic mice expressing mutant tau protein [58]. Nrf2-KO transgenic mice carrying both mutated APP and tau died prematurely (approximately 12 months of age) and were characterized by motor deficits, neuroinflammation [59], increased oxidative stress, and high levels of Aβ and tau aggregates [60].

A decrease in expression or inactivation of the p62/SQTRSM1 gene in mice causes neurodegeneration with AD-related symptoms and induces the formation of neurofibrillary tangles [61]. GSK3β is also involved in AD pathology since its hyperactivation has been demonstrated in AD brains [62], and GSK3β-mediated phosphorylation of PSN1 reduces neuronal viability and synaptic plasticity [63]. All these data indicate that AD-critical features, i.e., Aβ and tau protein aggregation, and oxidative stress are deeply related and cooperate in the development of pathology (Figure 3).

## 3. Small Molecules Inducing Nrf2

So far, the preclinical and clinical studies involving Nrf2 activators have mostly focused on cancer and inflammatory diseases therapies [5]. To date, the use of Nrf2 activators in neurological diseases is quite limited due to the unfavorable pharmacokinetic properties and toxicity issues of these compounds [5]. In the very last few years, advancements have been made in the research of new small-molecular Nrf2 activators endowed with CNS activity, which can be broadly classified into two distinct groups: (1) electrophilic activators that can covalently modify Keap1; (2) activators interfering with the Keap1–Nrf2 protein–protein interactions (PPIs) (Table 1).

### 3.1. Electrophilic Activators

As previously described, Keap1 ensures that Nrf2 concentration is kept very low thanks to oxidative stress sensors; however, if the oxidative stress increases, the ubiquitination and subsequent degradation of Nrf2 are blocked as a result of the reaction of electrophilic species with the cysteine residue in Keap1 protein [64]. According to this mechanism of induction of Nrf2, the use of electrophiles as drugs able to activate Nrf2 has gained increasing scientific interest, and, in the last few years, many electrophiles able to trigger the Nrf2 pathway have been reported. For example, molecules containing α,β-unsaturated ketones and esters have been shown to react with one or more cysteine residues in Keap1 [65,66], activating Nrf2. Indeed, these compounds are Michael acceptors, and they can undergo the nucleophilic addition of an alkyl thiol to their α,β-unsaturated system.

**Figure 4:**
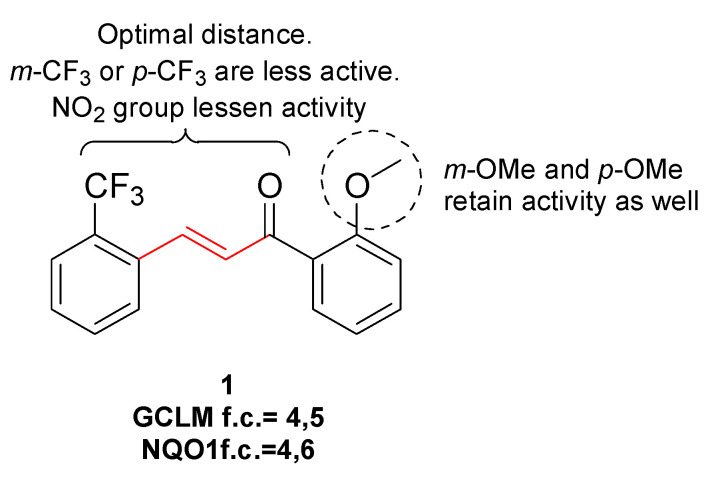
Compound **1**’s chemical structure and activity. The electrophilic α,β-unsaturated moiety of **1** is in red. This compound enhanced the expression of the antioxidant genes GCLM and NADPH-NQO1. The increase is reported as the relative fold change (f.c.) [68].

Chalcone contains an electrophilic α,β-unsaturated ketone that can react with the sulfhydryl groups of thioredoxin and cysteine residues in proteins [67]. Therefore, the α,β-unsaturated ketone electrophilic activity is responsible for the various biological activities of chalcones. One of the very first structure–activity relationship (SAR) studies on chalcone-based structures able to activate Nrf2 was reported in 2011 by Kumar et al. [68]. A series of 59 chalcone derivatives was synthesized, and compound **1** emerged as the most interesting molecule (Figure 4).

**Figure 5:**
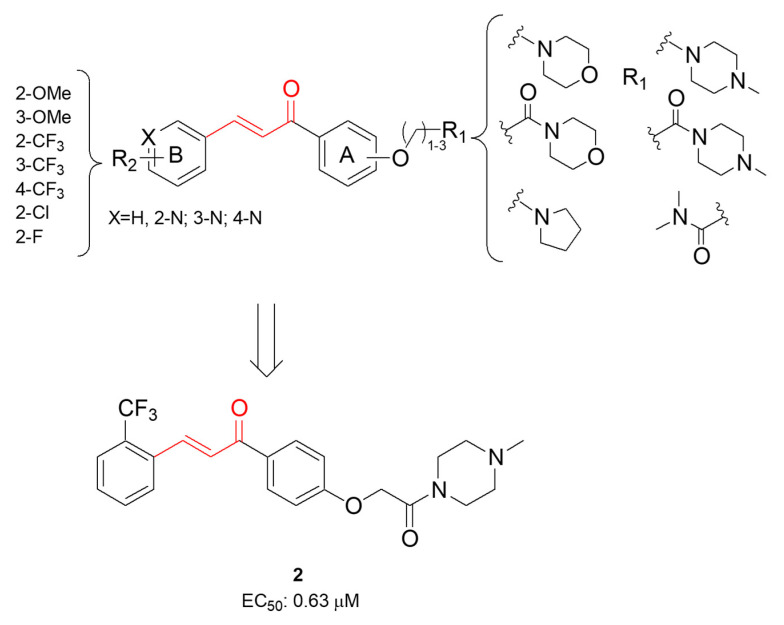
Novel chalcone derivatives as Nrf2 activators. The electrophilic α,β-unsaturated ketone moiety of the chalcone leading scaffold is in red. The introduction of a N-methylpiperazine group into the 4-OH of ring A led to compound **2,** a potent Nrf2 activator (EC_50_ = 0.63 μM) [69].

On the basis of this promising finding, the chalcone leading scaffold, i.e., the 1,3-diphenylprop-2-en-1-one core structure, was recently further investigated including heterocyclic amines such as morpholine, pyrrolidine, and N-methylpiperazine (Figure 5) [69]. In particular, the introduction of a propylmorphline group into the 2-, 3-, and 4-OH of ring A gave interesting results, especially for the 4-position substituted derivatives [69]. An additional enhancement in the Nrf2 activation was observed by replacing the 4-hydroxyl-propylmorpholine moiety with a 4-hydroxyl-propylpyrrolidine one. In addition, it was found that 2′-position substituted derivatives exhibited better activities than the corresponding 3′- and 4′-position substituted ones. The introduction of a N-methylpiperazine group into the 4-OH of ring A led to compound **2** (Figure 5), which showed the best Nrf2-activating potency in this series of compounds, resulting in an amelioration with respect to sulforaphane (SFN), a well-known strong Nrf2 activator (**2** EC_50_ = 0.63 μM vs. SFN EC_50_ = 0.87 μM) [69]. Compound **2** was evaluated in BV-2 microglial cells in both normal and proinflammatory conditions, where it stimulated the expression of Nrf2-dependent antioxidant enzymes and the reduction of ROS, inhibiting inflammatory responses induced by the LPS. Moreover, compound **2** was in vivo studied in the scopolamine-induced amnesia model, demonstrating the ability to restore SOD activity and memory performance in mice.

**Figure 6:**
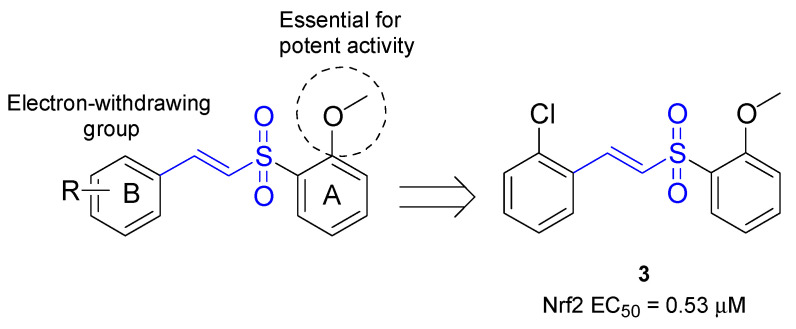
First generation of vinyl-sulfone-based Nrf2 activators. The vinyl-sulfone moiety is in blue. From the structure–activity relationships (SAR) study, it emerged that the presence of an electron-withdrawing group on the B ring favored the Nrf2 activation, while the presence of a 2-methoxyl group on the A ring was essential for the activity. Compound **3** emerged as the most active compound [70].

In another study, the structure of compound **1** was modified by introducing a vinyl-sulfoxide or vinyl-sulfone group to the α,β- unsaturated carbonyl entity of chalcone (Figure 6) [70].

**Figure 7:**
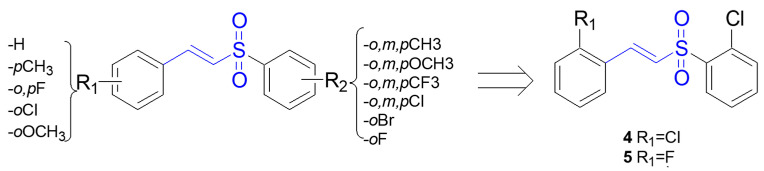
Optimization of vinyl-sulfone-based Nrf2 activators. The vinyl-sulfone moiety is in blue. Tuning the electrophilicity and steric hindrance of the aromatic substituents led to new Nrf2 activators. Compounds **4** and **5** were the most promising molecules of this series [71].

The introduction of the vinyl sulfone group led to the most potent activities in terms of HO-1 activation compared to the corresponding carbonyl and sulfoxide derivatives. On the basis of its potency of action, compound **3** was selected for further evaluations in DAergic neuronal cells, where it activated Nrf2 as expected, as well as stimulated the expression of the Nrf2-dependent antioxidant enzymes NQO1, GCLC, GLCM, and HO-1. Moreover, compound **3** was evaluated both in vitro and in MPTP-induced in vivo models of PD, demonstrating the attenuation of PD-associated behavioral deficits in the mouse model [70]. However, this molecule was not further investigated due to its poor pharmacokinetic properties; indeed, it showed low solubility, metabolic instability, and toxicity issues, such as cytochrome P (CYP) inhibition and human ether-a-go-go-related gene (hERG) activation. Recently, the molecular structural determinants responsible for vinyl-sulfone derivatives as Nrf2 activators endowed with neuroprotective activity on PC12 cells were investigated via the synthesis of a library of 47 small compounds (Figure 7) [71].

**Figure 8:**
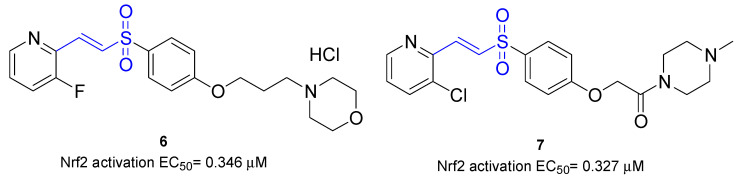
Chemical structure of vinyl-sulfone-based Nrf2 activators with improved activity and drug-like properties. The vinyl-sulfone moiety is in blue. The introduction of both a pyridyl group in the B ring and a morpholinyl or piperazinyl group in the A ring improved the microsomal stability and solubility of these compounds [72].

This study found that best results in terms of neuroprotection were obtained by molecules bearing aromatic *o*-substituents, and that, by tuning their electrophilicity and steric hindrance, potent Nrf2 activators can be obtained. In particular, the most interesting compounds of the considered series were **4** and **5** (Figure 7), which protected PC12 cells from H_2_O_2_-mediated insults, promoting the translocation of Nrf2 and the consequent activation of ARE, with the final transcriptional activation of antioxidant genes and the consequent upregulation of a diverse range of antioxidant species (TrxR, NQO1, GSH, and HO-1). Some years later, in order to improve the druglike properties of compound **3**, the B phenyl-ring was replaced by a pyridyl-moiety, while a morpholinyl group was linked to ring A to improve microsomal stability and increase solubility through the HCl salt form (**6,** Figure 8) [72].

**Figure 9:**
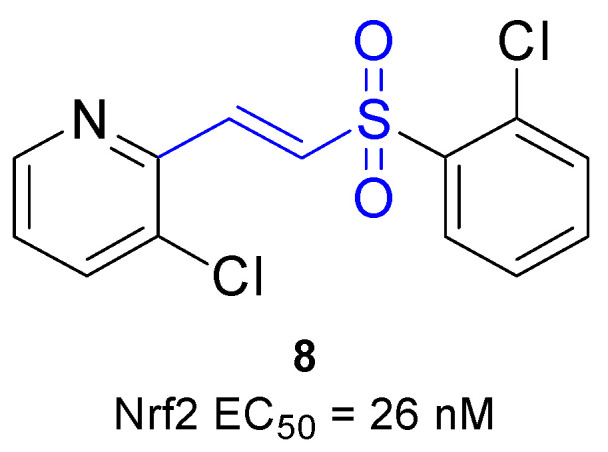
Chemical structure of a potent halogenated vinyl-sulfone-based Nrf2 activator. The vinyl-sulfone moiety is in blue. Compound **8** showed potent Nrf2 activation (EC_50_ = 26 nM) and drug-like properties [73].

Moreover, considering the potency of morpholine compounds with 3′-F or 3′-Cl on *o*-pyridine, a 4-piperazine group was introduced instead of the morpholino one (**7**, Figure 8). Compound **6** demonstrated excellent Nrf2 activation potency, safety, and stability, as well as elicited neuroprotective effects against DAergic neuronal cell death in PD, suppressing microglial activation related to neuroinflammation [72]. In a subsequent study, a series of pyridyl-vinyl-sulfones bearing a halogenated A-ring was synthesized in an attempt to maximize the potency of action of this class of Nrf2 activators [73]. Compound **8** (Figure 9) emerged as the most promising derivative, being able to activate Nrf2 at nanomolar concentrations, with an ameliorated potency of action with respect to the previous developed compounds. Moreover, compound **8** retained positive drug-like properties and an interesting biological profile in both in vitro and in vivo models of PD [73].

### 3.2. PPI Interfering Compounds: Azole-Based Compounds

**Figure 10:**
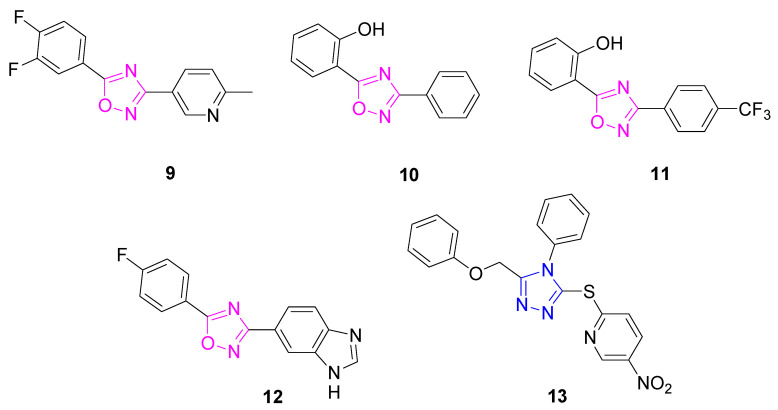
Oxadiazole- and triazole-based Nrf2 activators. The oxadiazole moiety is reported in pink, while the triazole moiety is in blue. These compounds were able to activate Nrf2 nuclear translocation [75,76,77,78].

**Figure 11:**
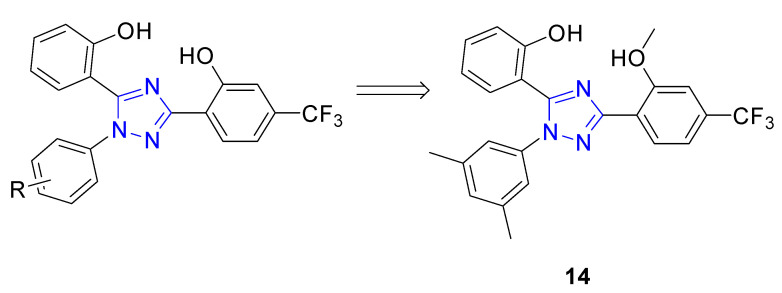
Development of 1,2,4-triazole derivatives as Nrf2 activators. The triazole moiety is in blue. The presence of alkyl groups on the diaryl-triazole scaffold was important for the activity, and **14** emerged as a potential neuroprotectant for the treatment of cerebral ischemic injury [79].

Interference with the protein–protein interaction between the KEAP1 Kelch domain and Nrf2 by means of non-electrophilic noncovalent inhibitors is increasingly being explored as an innovative approach to generate compounds activating Nrf2. These molecules are predicted to be safer with respect to electrophilic compounds, whose action is often associated with side-effects due to off-target actions, derived from their simultaneous reaction with different types of nucleophiles commonly present in many biological molecules. Several promising PPI inhibitors have been disclosed to date that are able to enhance Nrf2 signaling with different potential therapeutic applications [74], and diaryl-azole-based compounds in particular have been demonstrated as useful agents in neurological diseases. Starting from oxadiazole derivatives **9**–**12** (Figure 10) which were able to increase Nrf2 nuclear translocation up to 3.46-fold at 100 nM [75,76], similar triazole derivatives were designed, and compound **13** (Figure 10) was the most interesting one, showing promising results as neuroprotective agents [77,78]. In this compound, the chemical reactivity of the S-group with cysteine nucleophiles is essential for Nrf2 activation, as well as the presence of an electron-withdrawing group and an electron-deficient aromatic system. Very recently, diaryl-triazole derivatives were further investigated as Nrf2 activators able to counteract neuroinflammation. In particular, based on the leading scaffold of compound **11,** i.e., the diaryl-oxadiazole core structure (Figure 10), a library of 26 triazole-based compounds was synthesized (Figure 11), and the in vitro evaluation revealed that these molecules exerted better neuroprotective effects than **11** [79]. From the SAR analysis it emerged that alkyl groups were important to augment the protective effect; in particular, compound **14** (Figure 11) gave the best results. From the in-depth biological evaluation of this molecule, it emerged as a potential neuroprotectant for the treatment of ischemic stroke [79].

**Figure 12:**
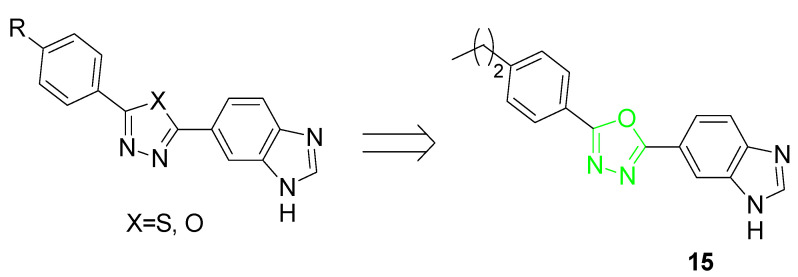
Nrf2 activators with a 1,3,4-oxa/thiadiazole core. The 1,3,4-oxadiazole moiety of compound **15** is in green. This molecule increased the Nrf2 levels and protected PC-12 cells from oxidative damage [80].

Further modifications of compound’s **11** azole core led to a series of 1,3,4-oxadiazole or 1,3,4-thiadiazole derivatives (Figure 12), displaying a potency of action as Nrf2 activators similar to the 1,2,4-oxadiazole derivatives (Figure 10) [80]. On the contrary, physicochemical properties were improved, since the presence of the 1,3,4-oxadiazole moiety led to lower lipophilicity, and it improved the aqueous solubility of these molecules. Compound **15** (Figure 12), displaying 1.8-fold induction of Nrf2 in a luciferase reporter assay at 100 nM, was selected for further evaluation. It was able to increase Nrf2 levels and its downstream genes in H_2_O_2_-treated PC-12 cells, protecting them from oxidative damage.

**Figure 13:**
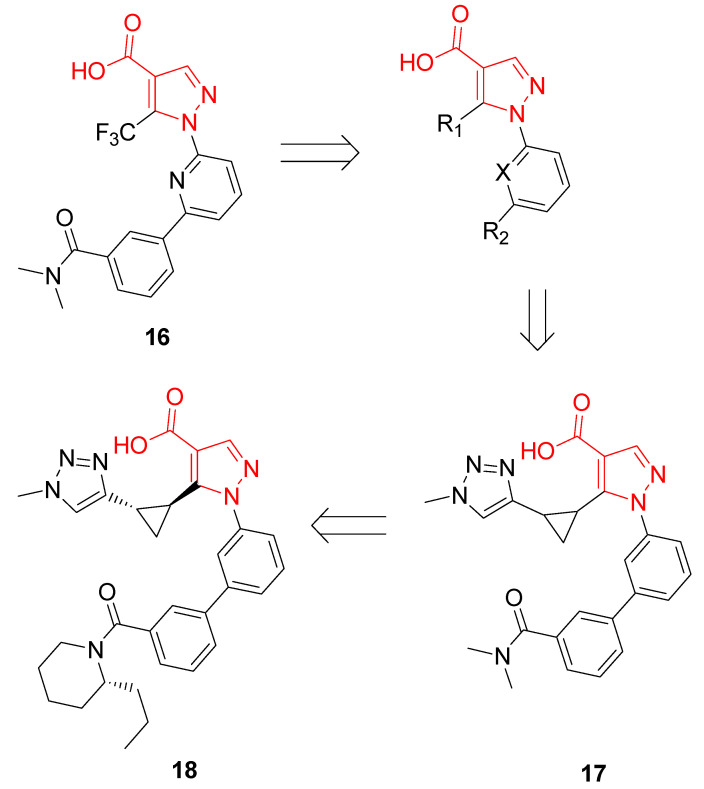
Development of pyrazole carboxylic acid inhibitors of the Keap1/Nrf2 complex protein–protein interactions. The pyrazole-carboxylic acid moiety is in red. Starting from the leading scaffold of **16**, i.e., the pyridyl-pyrazole carboxylic acid core structure, the triazole-cyclopropyl-derivative **17** was developed, showing a potent Nrf2 activation. This compound was further optimized by introducing a larger amide group, and the Nrf2 activator **18** was obtained, which was able to bind the Keap1 Kelch domain with increased affinity [81].

Very recently, pyrazole hit compounds as Keap1/Nrf2 complex disruptors were reported by means of a fragment-guided discovery approach [81]. Three “hotspots” for binding were identified in the Nrf2 binding site, i.e., for acidic groups, aromatic systems with a hydrogen bond acceptor (“planar acceptors”), and sulfonamides. From the screening of a collection of carboxylic acid derivatives, compound **16** (Figure 13) was selected for further optimization. The triazole-cyclopropyl derivative **17** then emerged as an interesting derivative, showing an IC_50_ of 41 nM in the displacement of the Nrf2 peptide from the KEAP1 Kelch protein fluorescence polarization assay. However, due to its high polarity, it failed the subsequent cell-based assay. Therefore, its structure was further optimized by introducing a larger amide moiety able to interact with lipophilic areas of the protein, and compound **18** was finally disclosed as the most promising agent (IC_50_ < 15 nM). Its high-affinity binding to the Kelch domain of KEAP1 was also confirmed by surface plasmon resonance (SPR) evaluation, with Kd = 2.5 nM [81]. The docking analysis of compound **18** in complex with KEAP1 showed that all the three hotspots identified from the initial fragment screen were occupied by the inhibitor.

**Table 1:** Main activity of the Nrf2 inducers discussed in Section 3. EC_50_ is the half maximal effective concentration.

Compound	Activity	Cell Line	Ref.
**2**	Activation of Nrf2 nuclear translocation (EC_50_ = 0.63 μM)	BV-2 microglial cells	[68]
**3**	Activation of Nrf2 nuclear translocation (EC_50_ = 0.53 μM)	CATH.a, mouse DAergic neuronal cell line	[70]
**4**, **5**	Activation of Nrf2 nuclear translocation; transcriptional activation of NRF2-responsive ARE genes	PC12 cell line	[71]
**6**	Activation of Nrf2 nuclear translocation (EC_50_ = 0.346 μM)	BV-2 microglial cells	[72]
**7**	Activation of Nrf2 nuclear translocation (EC_50_ = 0.327 μM)	BV-2 microglial cells	[72]
**8**	Activation of Nrf2 nuclear translocation (EC_50_ = 0.327 μM)	U2OS cells and BV-2 microglial cells	[73]
**13**	Induction of NQO1 and GCLM proteins; transcriptional activation of NRF2-responsive ARE genes	Primary mouse neurons; primary corticostriatal neuronal cocultures	[77,78]
**14**	Activation of Nrf2 nuclear translocation; induction of HO-1, NQO1, and GCLM proteins; Nrf2 displacement from the Keap1 Kealch domain (K_D_ = 22.70 μM)	PC12 cell line	[79]
**15**	ARE inducing activity fold increase (f.i. = 1.8 @100 nM)	PC12 cell line	[80]
**18**	Stimulation of NQO1 activity (EC_50_= 43 nM); Nrf2 displacement from the Keap1 Kealch domain (IC_50_ < 15 nM); transcriptional activation of NRF2-responsive ARE genes	BEAS-2B cells; normal human bronchial epithelial cells	[81]

## 4. Natural Nrf2 Activators

**Figure 14:**
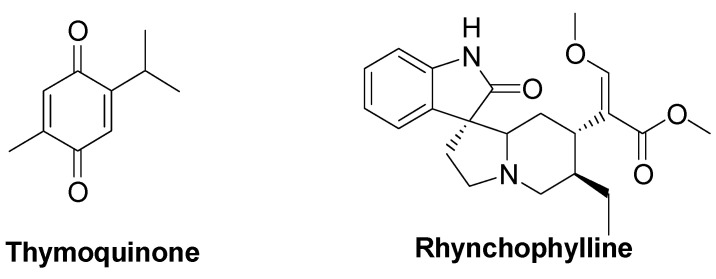
Chemical structure of natural Nrf2 activators with neuroprotectant activity. Thymoquinone prevents dopaminergic neurodegeneration in a PD mouse model, activating the nuclear translocation of Nrf2 for binding to ARE [85]. Rhynchophylline has shown neuroprotective effects in animal models of AD, as well as antioxidant properties [87,88,89].

Thymoquinone (TQ, 2-isopropyl-5-methyl-1, 4-benzoquinone, Figure 14) is a monoterpenoid hydrocarbon, and it is the major bioactive compound of the volatile black oil from Nigella sativa. This molecule is endowed with antioxidant and anti-inflammatory properties [82], in addition to counteracting apoptosis in primary dopaminergic cells after their treatment with 1-methyl-4-phenylpyridinium (MPP+) and rotenone [83,84]. Recently, it was found that TQ prevents dopaminergic neurodegeneration in a PD mouse model, activating the nuclear translocation of Nrf2 for binding to ARE. As a consequence, the induction of HO-1, NQO1, and GST expression, as well as of anti-oxidative enzymes, including SOD and GSH-Px, was observed [85]. From a mechanistic viewpoint, TQ is an electrophilic activator that covalently binds the thiol group of the KEAP1 protein, which is the main target of TQ when its biological effects are mediated by Nrf2 [86].

Uncaria rhynchophylla (UR) is a component of the traditional Japanese kampo medicines chotosan and yokukansan, and rhynchophylline (Rhy, Figure 14) is a primary oxindole alkaloid obtained from UR. This natural compound has shown neuroprotective effects in animal models of AD, as well as antioxidant properties [87,88,89]. In an in vivo model of Aβ1–42-induced AD, Rhy was able to attenuate the disease symptoms and the in vitro investigations in SH-SY5Y cells revealed that this compound activates Nrf2 nuclear translocation, triggering the expression of its downstream antioxidant enzymes. However, how Rhy activates the Nrf2–ARE pathway to exert neuroprotection remains to be clarified [90].

## 5. Multitargeting Nrf2 Activators

Because of the multifactorial etiology of neurodegenerative diseases, in the last few years, the design of multifunctional ligands has emerged as an attractive therapeutic strategy. This multifactorial approach, involving the use of multitarget-directed ligands (MTDL), certainly reflects the enormous and crucial advances in understanding the mechanisms and implications in neurodegenerative diseases [91]. In this context, multitarget ligands have been developed very recently, exhibiting significant Nrf2 inducibility and an additional activity on other targets related to neurodegeneration.

**Figure 15:**
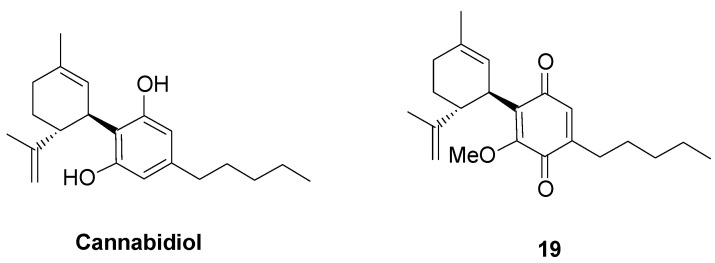
BACH1 inhibitor and Nrf2 inducer compounds. Chemical structure of the cannabidiol derivative **19** [92].

Considering the significance of both BACH1 and Nrf2 in the modulation of heme oxygenase 1 (HMOX1) expression, which protects cells from neurotoxicity [92], the combination of Nrf2 activation with BACH1 inhibition could potentially result in an antioxidant response and a neuroprotective outcome. Recently, the O-methyl-p-cannabidiolquinone (**19**, Figure 15), a derivative of cannabidiol, was found at the same time as a potent inhibitor of BACH1 and an Nrf2 activator.

These activities were validated in HaCaT and Hepa1c1c7 cell lines through luciferase assays, Western blot, and RT-qPCR [93]. Compound **19** was also tested in macrophage-like THP1 and neuroblastoma SH-SY-5Y cell lines, confirming a decrease in BACH1, an induction of HMOX1, and a stabilization of Nrf2, as well as a decrease in ROS levels and neuroprotection in a model for Huntington disease. In terms of molecular structure, it is likely that compound **19** contributed to the regulation of redox homeostasis of cells through its quinone moieties with antioxidant properties [94].

**Figure 16:**
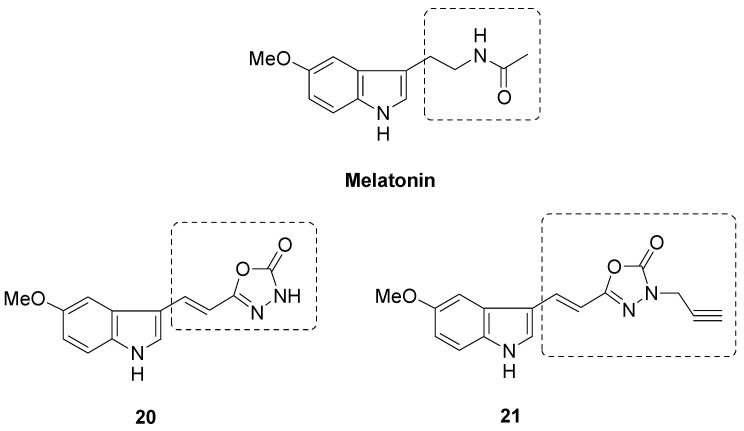
QR2 inhibitor and Nrf2 inducer dual agents. Chemical structures of the melatonin derivatives **20** and **21** [96].

Melatonin is a hormone that binds the G protein-coupled receptors MT1 and MT2, as well as the cytosolic receptor MT3/quinone reductase 2 (QR2). QR2 catalyzes the reduction of electrophilic quinones to hydroquinones, which can trigger an overproduction of free radicals. For this reason, this enzyme can be considered a molecular target involved in neurotoxic cascades [95]. Within the framework of a study of oxadiazolone-based bioisosteres of melatonin, two compounds (**20** and **21**, Figure 16) were developed, showing an induction of signaling mediated by Nrf2 (induction capability of 15.1 and 1.76, respectively) and an inhibition of QR2 (Ki 6.6 nM and 3.2 nM, respectively) in a neuronal phenotype [96].

Interestingly, the absorption, distribution, metabolism, excretion, and toxicity (ADMET) and antioxidant properties of these compounds, also evaluated with the oxygen radical absorbance capacity (ORAC) assay, were satisfactory [96]. Quantum mechanics studies revealed that the ring systems contained in the indole-NH-oxadiazolone **20** are coplanar. This molecular arrangement is involved in the stacking interaction with the flavin adenine dinucleotide (FAD) in QR2, allowing the indole nitrogen to establish an interaction with the carbonyl oxygen of Gly174, reminiscent of that observed for polyphenol hydroxyls. Moreover, molecular docking studies and molecular dynamics simulations showed that compounds **20** and **21** bind to the KEAP1 Kelch domain, and their indole core and double bond establish important interactions with essential residues involved in the binding with Nrf2.

**Figure 17:**
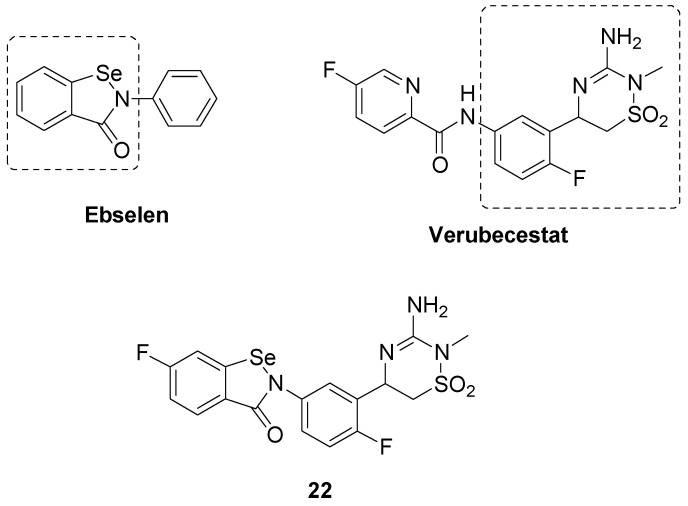
BACE-1 inhibitor and Keap1–Nrf2–ARE pathway activator. Hybridization of ebselen and verubecestat led to **22**, a candidate for the treatment of AD [99].

Beta-site amyloid precursor protein (APP)-cleaving enzyme 1 (BACE1) is an aspartyl protease widely expressed in the brain, essential for the synthesis of monomeric amyloid-β (Aβ), which spontaneously self-aggregates to form the insoluble fibers known as senile plaques, initiating toxicity in AD [97]. BACE1 is considered a well-validated therapeutic target for AD, and this approach was used for obtaining potent BACE1 inhibitors, such as verubecestat (MK8931, Figure 17) [98]. Within a series of selenium-containing compounds, including the key pharmacophores of verubecestat and ebselen (a potent antioxidant activating the Nrf2 pathway, Figure 17), the derivative **22** (Figure 17) was selected as a promising multifunctional candidate for pharmacological therapy of AD [99]. Biological evaluation showed that compound **22** exhibited good BACE-1 inhibition and Keap1–Nrf2–ARE pathway activation. Furthermore, it alleviates oxidative cell damage induced by H_2_O_2_ or 6-OHDA, reduced Aβ1-40 in HEK APPswe 293T cells, and was able to cross the BBB.

In the context of AD, another multitarget approach was achieved through the preparation of merged ligands with dual pharmacological activity and subsequent structural optimization [100].

**Figure 18:**
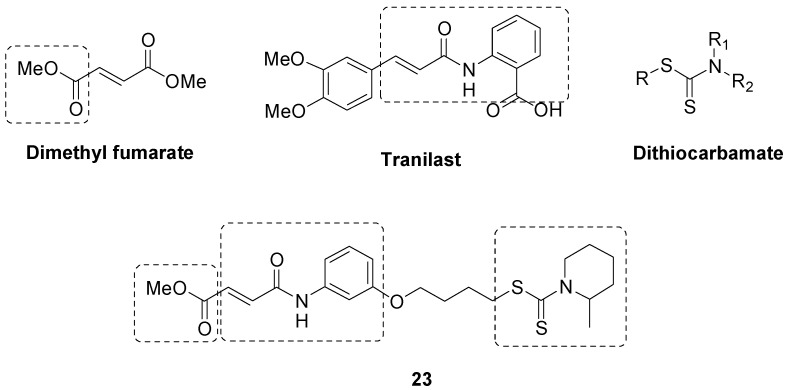
AChE inhibitor and Keap1–Nrf2–ARE pathway activator. Chemical structure of the candidate for the treatment of AD **23**, which resulted from the combination of different pharmacophoric elements [103].

The cholinergic hypothesis of AD suggests that the cognitive decline observed in the disease can be attributed to a selective loss of cholinergic neurons of the basal forebrain. Acetylcholinesterase (AChE) hydrolyzes acetylcholine (ACh), terminating the cholinergic transmission. AChE inhibitors, considered in the therapeutic armamentarium of AD, prevent the degradation of the neurotransmitter, allowing to prolong the action of the deficient neurotransmitter in the brain [101]. Three scaffolds were combined to form a series of molecules containing a flexible carbon chain: (i) dimethyl fumarate, containing an α,β-unsaturated ketone, a representative structure for Nrf2 activation, (ii) tranilast, an old antiallergic drug with anti-inflammatory properties patented for treating AD [102], and (iii) dithiocarbamate, able to interact with the catalytic active site of AChE. Among the tested derivatives, the most potent inhibitor of human AChE was compound **23** (Figure 18, IC_50_ 53 nM) which also showed activation of the Keap1–Nrf2–ARE pathway, antioxidant and anti-inflammatory effects, and permeation of the BBB [103]. From in vivo studies, it emerged that it ameliorated cognitive deficits in the scopolamine-induced mouse model of AD [103].

**Figure 19:**
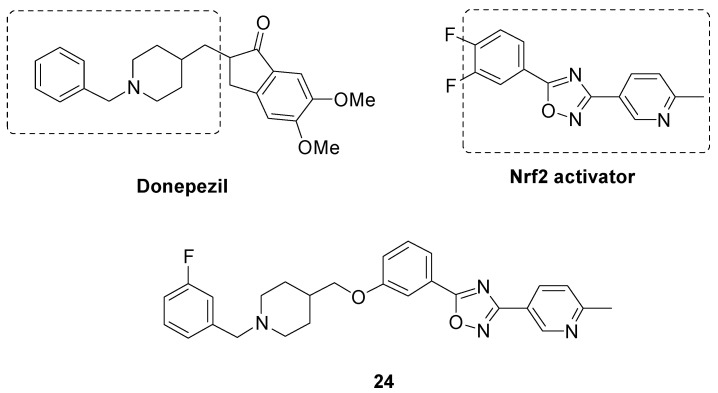
AChE inhibitor and Nrf2 activator dual agent. Chemical structure of the hybrid donepezil/1,2,4-oxadiazole compound **24**, a candidate for the treatment of AD [75].

A further combination of Nrf2 activator and AChE inhibitor was recently proposed, characterized by the presence of a 1,2,4-oxadiazole core and benzylpiperidine [103]. Among the various derivatives synthesized, compound **24** was selected as the representative molecule (Figure 19).

It exhibited excellent human AChE inhibition (hAChE IC_50_ = 0.38 μM) in an ARE luciferase reporter test and it was not cytotoxic against PC12 cells. The inhibitory AchE was ascribed to the benzylpiperidine motif from the AChE inhibitor donepezil, which fits into in the catalytic anionic site of enzyme. Compound **24** also presented Nrf2-inducing activity, determined with an ARE-luciferase reporter assay, as well as an upregulation effect on downstream proteins HO-1, NQO1, and GCLM, with significant antioxidative and anti-inflammatory potency. The 1,2,4-oxadiazole core was identified to have inductivity of Nrf2 [75]. The administration of this bifunctional agent led to a more marked improvement in cognition and inflammation than the combination of an AChE inhibitor and an Nrf2 activator.

## 6. Conclusions

The potential clinical application of Nrf2 activators has been widely reported for the treatment of different diseases, basically having in common oxidative stress and chronic inflammation as hallmarks. However, in very recent years, the interest in the potential therapeutic role of Nrf2 activators for the treatment of neurodegenerative diseases has grown, and several small molecules have been disclosed, also aiming toward a polypharmacologic approach, based on the multifactorial etiology of these pathological conditions. In this review, we reported the latest and most important findings in the research on compounds able to upregulate the Keap1–Nrf2–ARE pathway, considering activators, Nrf2/Keap1 protein–protein degraders, and multitargeting compounds, all showing a usefulness in counteracting neurodegeneration. The drug design of the reported molecules and their activity have been discussed, with a focus on the amelioration of their pharmacokinetic parameters. In fact, despite the many promising results shown by Nrf2 activators, their clinical use has been limited by issues regarding the interaction with off-targets and their lack of selectivity, as well as by poor BBB penetration [5]. All in all, although the future of Nrf2 activators seems promising, more research is necessary in this field to translate these compounds into the clinical use.

## Data Availability

Data sharing not applicable.
